# Virus-Vectored Influenza Virus Vaccines

**DOI:** 10.3390/v6083055

**Published:** 2014-08-07

**Authors:** Ralph A. Tripp, S. Mark Tompkins

**Affiliations:** Department of Infectious Diseases, University of Georgia, 111 Carlton St., Athens, GA 30602, USA; E-Mail: smt@uga.edu

**Keywords:** influenza virus, vaccine, recombinant vaccine, virus vector, universal vaccine

## Abstract

Despite the availability of an inactivated vaccine that has been licensed for >50 years, the influenza virus continues to cause morbidity and mortality worldwide. Constant evolution of circulating influenza virus strains and the emergence of new strains diminishes the effectiveness of annual vaccines that rely on a match with circulating influenza strains. Thus, there is a continued need for new, efficacious vaccines conferring cross-clade protection to avoid the need for biannual reformulation of seasonal influenza vaccines. Recombinant virus-vectored vaccines are an appealing alternative to classical inactivated vaccines because virus vectors enable native expression of influenza antigens, even from virulent influenza viruses, while expressed in the context of the vector that can improve immunogenicity. In addition, a vectored vaccine often enables delivery of the vaccine to sites of inductive immunity such as the respiratory tract enabling protection from influenza virus infection. Moreover, the ability to readily manipulate virus vectors to produce novel influenza vaccines may provide the quickest path toward a universal vaccine protecting against all influenza viruses. This review will discuss experimental virus-vectored vaccines for use in humans, comparing them to licensed vaccines and the hurdles faced for licensure of these next-generation influenza virus vaccines.

## 1. Introduction

Seasonal influenza is a worldwide health problem causing high mobility and substantial mortality [1,2,3,4]. Moreover, influenza infection often worsens preexisting medical conditions [5,6,7]. Vaccines against circulating influenza strains are available and updated annually, but many issues are still present, including low efficacy in the populations at greatest risk of complications from influenza virus infection, *i.e.*, the young and elderly [8,9]. Despite increasing vaccination rates, influenza-related hospitalizations are increasing [8,10], and substantial drug resistance has developed to two of the four currently approved anti-viral drugs [11,12]. While adjuvants have the potential to improve efficacy and availability of current inactivated vaccines, live-attenuated and virus-vectored vaccines are still considered one of the best options for the induction of broad and efficacious immunity to the influenza virus [13].

The general types of influenza vaccines available in the United States are trivalent inactivated influenza vaccine (TIV), quadrivalent influenza vaccine (QIV), and live attenuated influenza vaccine (LAIV; in trivalent and quadrivalent forms). There are three types of inactivated vaccines that include whole virus inactivated, split virus inactivated, and subunit vaccines. In split virus vaccines, the virus is disrupted by a detergent. In subunit vaccines, HA and NA have been further purified by removal of other viral components. TIV is administered intramuscularly and contains three or four inactivated viruses, *i.e.*, two type A strains (H1 and H3) and one or two type B strains. TIV efficacy is measured by induction of humoral responses to the hemagglutinin (HA) protein, the major surface and attachment glycoprotein on influenza. Serum antibody responses to HA are measured by the hemagglutination-inhibition (HI) assay, and the strain-specific HI titer is considered the gold-standard correlate of immunity to influenza where a four-fold increase in titer post-vaccination, or a HI titer of ≥1:40 is considered protective [4,14]. Protection against clinical disease is mainly conferred by serum antibodies; however, mucosal IgA antibodies also may contribute to resistance against infection. Split virus inactivated vaccines can induce neuraminidase (NA)-specific antibody responses [15,16,17], and anti-NA antibodies have been associated with protection from infection in humans [18,19,20,21,22]. Currently, NA-specific antibody responses are not considered a correlate of protection [14]. LAIV is administered as a nasal spray and contains the same three or four influenza virus strains as inactivated vaccines but on an attenuated vaccine backbone [4]. LAIV are temperature-sensitive and cold-adapted so they do not replicate effectively at core body temperature, but replicate in the mucosa of the nasopharynx [23]. LAIV immunization induces serum antibody responses, mucosal antibody responses (IgA), and T cell responses. While robust serum antibody and nasal wash (mucosal) antibody responses are associated with protection from infection, other immune responses, such as CD8^+^ cytotoxic lymphocyte (CTL) responses may contribute to protection and there is not a clear correlate of immunity for LAIV [4,14,24].

Currently licensed influenza virus vaccines suffer from a number of issues. The inactivated vaccines rely on specific antibody responses to the HA, and to a lesser extent NA proteins for protection. The immunodominant portions of the HA and NA molecules undergo a constant process of antigenic drift, a natural accumulation of mutations, enabling virus evasion from immunity [9,25]. Thus, the circulating influenza A and B strains are reviewed annually for antigenic match with current vaccines, Replacement of vaccine strains may occur regularly, and annual vaccination is recommended to assure protection [4,26,27]. For the northern hemisphere, vaccine strain selection occurs in February and then manufacturers begin production, taking at least six months to produce the millions of vaccine doses required for the fall [27]. If the prediction is imperfect, or if manufacturers have issues with vaccine production, vaccine efficacy or availability can be compromised [28]. LAIV is not recommended for all populations; however, it is generally considered to be as effective as inactivated vaccines and may be more efficacious in children [4,9,24]. While LAIV relies on antigenic match and the HA and NA antigens are replaced on the same schedule as the TIV [4,9], there is some suggestion that LAIV may induce broader protection than TIV due to the diversity of the immune response consistent with inducing virus-neutralizing serum and mucosal antibodies, as well as broadly reactive T cell responses [9,23,29]. While overall both TIV and LAIV are considered safe and effective, there is a recognized need for improved seasonal influenza vaccines [26]. Moreover, improved understanding of immunity to conserved influenza virus antigens has raised the possibility of a universal vaccine, and these universal antigens will likely require novel vaccines for effective delivery [30,31,32].

## 2. Virus-Vectored Vaccines

Virus-vectored vaccines share many of the advantages of LAIV, as well as those unique to the vectors. Recombinant DNA systems exist that allow ready manipulation and modification of the vector genome. This in turn enables modification of the vectors to attenuate the virus or enhance immunogenicity, in addition to adding and manipulating the influenza virus antigens. Many of these vectors have been extensively studied or used as vaccines against wild type forms of the virus. Finally, each of these vaccine vectors is either replication-defective or causes a self-limiting infection, although like LAIV, safety in immunocompromised individuals still remains a concern [4,13,33,34,35]. Table 1 summarizes the benefits and concerns of each of the virus-vectored vaccines discussed here. 

### 2.1. Adenovirus Vectors

There are 53 serotypes of adenovirus, many of which have been explored as vaccine vectors. A live adenovirus vaccine containing serotypes 4 and 7 has been in use by the military for decades, suggesting adenoviruses may be safe for widespread vaccine use [36]. However, safety concerns have led to the majority of adenovirus-based vaccine development to focus on replication-defective vectors. Adenovirus 5 (Ad5) is the most-studied serotype, having been tested for gene delivery and anti-cancer agents, as well as for infectious disease vaccines.

Adenovirus vectors are attractive as vaccine vectors because their genome is very stable and there are a variety of recombinant systems available which can accommodate up to 10 kb of recombinant genetic material [37]. Adenovirus is a non-enveloped virus which is relatively stable and can be formulated for long-term storage at 4 °C, or even storage up to six months at room temperature [33]. Adenovirus vaccines can be grown to high titers, exceeding 10^1^° plaque forming units (PFU) per mL when cultured on 293 or PER.C6 cells [38], and the virus can be purified by simple methods [39]. Adenovirus vaccines can also be delivered via multiple routes, including intramuscular injection, subcutaneous injection, intradermal injection, oral delivery using a protective capsule, and by intranasal delivery. Importantly, the latter two delivery methods induce robust mucosal immune responses and may bypass preexisting vector immunity [33]. Even replication-defective adenovirus vectors are naturally immunostimulatory and effective adjuvants to the recombinant antigen being delivered.

**Table 1:** Virus-vectored experimental influenza vaccines.

Vectored Vaccine	Benefits	Concerns
Adenovirus	Stable, readily manipulated vector system Replicates in established vaccine cell lines Can be dried; no cold chain required Robust transgene expression Infects a broad range of cell types Extensive safety record and clinical trial data Robust innate, cellular and humoral immune responses	Potential for replication-competent adenovirus (RCA) Preexisting immunity to common serotypes (Ad5) DNA genome raises concerns of integration Negative image from failed gene therapy and HIV vaccine trials (Ad5) Alternative (non-human and rare serotype) adenovirus vectors have limited efficacy and safety data
Adeno-Associated Virus	Limited preexisting immunity Limited/no cold chain	Limited safety data for humans Limited efficacy data
Alphavirus	RNA genome (should not modify host genome) Readily amenable to vaccine design Robust innate, cellular and humoral immune responses Licensed recombinant veterinary vaccine	Requires cold chain Limited safety data for humans
Baculovirus	Non-mammalian vector Established scale-up from recombinant protein expression programs	Limited efficacy data Safety in humans not established
Newcastle Disease Virus	RNA genome (should not modify host genome) Stable, readily manipulated vector system Replicates in established vaccine cell lines No preexisting immunity (avian virus)	Requires cold chain Limited safety data for humans
Parainfluenza Virus 5	RNA genome (should not modify host genome) Stable, readily manipulated vector system Avirulent in many species Replicates in some vaccine cell lines tested Preexisting immunity does not affect immunogenicity Robust innate, cellular and humoral immune responses	Requires cold chain Safety in humans not established Limited efficacy data
Poxvirus Vectors	Stable, readily manipulated vector system Replicates in established vaccine cell lines Can be dried; no cold chain required Extensive safety record and clinical trial data Robust innate, cellular and humoral immune responses	Preexisting immunity DNA genome raises concerns of integration
Vesicular Stomatitis	RNA genome (should not modify host genome) Replicates in established vaccine cell lines	Requires cold chain Safety in humans not established

Adenovirus has been extensively studied as a vaccine vector for human disease. The first report using adenovirus as a vaccine vector for influenza demonstrated immunogenicity of recombinant adenovirus 5 (rAd5) expressing the HA of a swine influenza virus, A/Swine/Iowa/1999 (H3N2). Intramuscular immunization of mice with this construct induced robust neutralizing antibody responses and protected mice from challenge with a heterologous virus, A/Hong Kong/1/1968 (H3N2) [40]. Replication defective rAd5 vaccines expressing influenza HA have also been tested in humans. A rAd5-HA expressing the HA from A/Puerto Rico/8/1934 (H1N1; PR8) was delivered to humans epicutaneously or intranasally and assayed for safety and immunogenicity. The vaccine was well tolerated and induced seroconversion with the intranasal administration had a higher conversion rate and higher geometric meant HI titers [41]. While clinical trials with rAd vectors have overall been successful, demonstrating safety and some level of efficacy, rAd5 as a vector has been negatively overshadowed by two clinical trial failures. The first trial was a gene therapy examination where high-dose intravenous delivery of an Ad vector resulted in the death of an 18-year-old male [42,43]. The second clinical failure was using an Ad5-vectored HIV vaccine being tested as a part of a Step Study, a phase 2B clinical trial. In this study, individuals were vaccinated with the Ad5 vaccine vector expressing HIV-1 gag, pol, and nef genes. The vaccine induced HIV-specific T cell responses; however, the study was stopped after interim analysis suggested the vaccine did not achieve efficacy and individuals with high preexisting Ad5 antibody titers might have an increased risk of acquiring HIV-1 [44,45,46]. Subsequently, the rAd5 vaccine-associated risk was confirmed [47]. While these two instances do not suggest Ad-vector vaccines are unsafe or inefficacious, the umbra cast by the clinical trials notes has affected interest for all adenovirus vaccines, but interest still remains.

Immunization with adenovirus vectors induces potent cellular and humoral immune responses that are initiated through toll-like receptor-dependent and independent pathways which induce robust pro-inflammatory cytokine responses. Recombinant Ad vaccines expressing HA antigens from pandemic H1N1 (pH1N1), H5 and H7 highly pathogenic avian influenza (HPAI) virus (HPAIV), and H9 avian influenza viruses have been tested for efficacy in a number of animal models, including chickens, mice, and ferrets, and been shown to be efficacious and provide protection from challenge [48,49]. Several rAd5 vectors have been explored for delivery of non-HA antigens, influenza nucleoprotein (NP) and matrix 2 (M2) protein [29,50,51,52]. The efficacy of non-HA antigens has led to their inclusion with HA-based vaccines to improve immunogenicity and broaden breadth of both humoral and cellular immunity [53,54]. However, as both CD8^+^ T cell and neutralizing antibody responses are generated by the vector and vaccine antigens, immunological memory to these components can reduce efficacy and limit repeated use [48].

One drawback of an Ad5 vector is the potential for preexisting immunity, so alternative adenovirus serotypes have been explored as vectors, particularly non-human and uncommon human serotypes. Non-human adenovirus vectors include those from non-human primates (NHP), dogs, sheep, pigs, cows, birds and others [48,55]. These vectors can infect a variety of cell types, but are generally attenuated in humans avoiding concerns of preexisting immunity. Swine, NHP and bovine adenoviruses expressing H5 HA antigens have been shown to induce immunity comparable to human rAd5-H5 vaccines [33,56]. Recombinant, replication-defective adenoviruses from low-prevalence serotypes have also been shown to be efficacious. Low prevalence serotypes such as adenovirus types 3, 7, 11, and 35 can evade anti-Ad5 immune responses while maintaining effective antigen delivery and immunogenicity [48,57]. Prime-boost strategies, using DNA or protein immunization in conjunction with an adenovirus vaccine booster immunization have also been explored as a means to avoided preexisting immunity [52].

### 2.2. Adeno-Associated Virus Vectors

Adeno-associated viruses (AAV) were first explored as gene therapy vectors. Like rAd vectors, rAAV have broad tropism infecting a variety of hosts, tissues, and proliferating and non-proliferating cell types [58]. AAVs had been generally not considered as vaccine vectors because they were widely considered to be poorly immunogenic. A seminal study using AAV-2 to express a HSV-2 glycoprotein showed this virus vaccine vector effectively induced potent CD8^+^ T cell and serum antibody responses, thereby opening the door to other rAAV vaccine-associated studies [59,60].

AAV vector systems have a number of engaging properties. The wild type viruses are non-pathogenic and replication incompetent in humans and the recombinant AAV vector systems are even further attenuated [61]. As members of the parvovirus family, AAVs are small non-enveloped viruses that are stable and amenable to long-term storage without a cold chain. While there is limited preexisting immunity, availability of non-human strains as vaccine candidates eliminates these concerns. Modifications to the vector have increased immunogenicity, as well [60].

There are limited studies using AAVs as vaccine vectors for influenza. An AAV expressing an HA antigen was first shown to induce protective in 2001 [62]. Later, a hybrid AAV derived from two non-human primate isolates (AAVrh32.33) was used to express influenza NP and protect against PR8 challenge in mice [63]. Most recently, following the 2009 H1N1 influenza virus pandemic, rAAV vectors were generated expressing the HA, NP and matrix 1 (M1) proteins of A/Mexico/4603/2009 (pH1N1), and in murine immunization and challenge studies, the rAAV-HA and rAAV-NP were shown to be protective; however, mice vaccinated with rAAV-HA + NP + M1 had the most robust protection. Also, mice vaccinated with rAAV-HA + rAAV-NP + rAAV-M1 were also partially protected against heterologous (PR8, H1N1) challenge [63]. Most recently, an AAV vector was used to deliver passive immunity to influenza [64,65]. In these studies, AAV (AAV8 and AAV9) was used to deliver an antibody transgene encoding a broadly cross-protective anti-influenza monoclonal antibody for *in vivo* expression. Both intramuscular and intranasal delivery of the AAVs was shown to protect against a number of influenza virus challenges in mice and ferrets, including H1N1 and H5N1 viruses [64,65]. These studies suggest that rAAV vectors are promising vaccine and immunoprophylaxis vectors. To this point, while approximately 80 phase I, I/II, II, or III rAAV clinical trials are open, completed, or being reviewed, these have focused upon gene transfer studies and so there is as yet limited safety data for use of rAAV as vaccines [66].

### 2.3. Alphavirus Vectors

Alphaviruses are positive-sense, single-stranded RNA viruses of the *Togaviridae* family. A variety of alphaviruses have been developed as vaccine vectors, including Semliki Forest virus (SFV), Sindbis (SIN) virus, Venezuelan equine encephalitis (VEE) virus, as well as chimeric viruses incorporating portions of SIN and VEE viruses. The replication defective vaccines or replicons do not encode viral structural proteins, having these portions of the genome replaces with transgenic material. The structural proteins are provided in cell culture production systems. One important feature of the replicon systems is the self-replicating nature of the RNA. Despite the partial viral genome, the RNAs are self-replicating and can express transgenes at very high levels [67].

SIN, SFV, and VEE have all been tested for efficacy as vaccine vectors for influenza virus [68,69,70,71]. A VEE-based replicon system encoding the HA from PR8 was demonstrated to induce potent HA-specific immune response and protected from challenge in a murine model, despite repeated immunization with the vector expressing a control antigen, suggesting preexisting immunity may not be an issue for the replicon vaccine [68]. A separate study developed a VEE replicon system expressing the HA from A/Hong Kong/156/1997 (H5N1) and demonstrated varying efficacy after *in ovo* vaccination or vaccination of 1-day-old chicks [70]. A recombinant SIN virus was use as a vaccine vector to deliver a CD8^+^ T cell epitope only. The well-characterized NP epitope was transgenically expressed in the SIN system and shown to be immunogenic in mice, priming a robust CD8^+^ T cell response and reducing influenza virus titer after challenge [69]. More recently, a VEE replicon system expressing the HA protein of PR8 was shown to protect young adult (8-week-old) and aged (12-month-old) mice from lethal homologous challenge [72].

The VEE replicon systems are particularly appealing as the VEE targets antigen-presenting cells in the lymphatic tissues, priming rapid and robust immune responses [73]. VEE replicon systems can induce robust mucosal immune responses through intranasal or subcutaneous immunization [72,73,74], and subcutaneous immunization with virus-like replicon particles (VRP) expressing HA-induced antigen-specific systemic IgG and fecal IgA antibodies [74]. VRPs derived from VEE virus have been developed as candidate vaccines for cytomegalovirus (CMV). A phase I clinical trial with the CMV VRP showed the vaccine was immunogenic, inducing CMV-neutralizing antibody responses and potent T cell responses. Moreover, the vaccine was well tolerated and considered safe [75]. A separate clinical trial assessed efficacy of repeated immunization with a VRP expressing a tumor antigen. The vaccine was safe and despite high vector-specific immunity after initial immunization, continued to boost transgene-specific immune responses upon boost [76]. While additional clinical data is needed, these reports suggest alphavirus replicon systems or VRPs may be safe and efficacious, even in the face of preexisting immunity.

### 2.4. Baculovirus Vectors

Baculovirus has been extensively used to produce recombinant proteins. Recently, a baculovirus-derived recombinant HA vaccine was approved for human use and was first available for use in the United States for the 2013–2014 influenza season [4]. Baculoviruses have also been explored as vaccine vectors. Baculoviruses have a number of advantages as vaccine vectors. The viruses have been extensively studied for protein expression and for pesticide use and so are readily manipulated. The vectors can accommodate large gene insertions, show limited cytopathic effect in mammalian cells, and have been shown to infect and express genes of interest in a spectrum of mammalian cells [77]. While the insect promoters are not effective for mammalian gene expression, appropriate promoters can be cloned into the baculovirus vaccine vectors.

Baculovirus vectors have been tested as influenza vaccines, with the first reported vaccine using Autographa californica nuclear polyhedrosis virus (AcNPV) expressing the HA of PR8 under control of the CAG promoter (AcCAG-HA) [77]. Intramuscular, intranasal, intradermal, and intraperitoneal immunization or mice with AcCAG-HA elicited HA-specific antibody responses, however only intranasal immunization provided protection from lethal challenge. Interestingly, intranasal immunization with the wild type AcNPV also resulted in protection from PR8 challenge. The robust innate immune response to the baculovirus provided non-specific protection from subsequent influenza virus infection [78]. While these studies did not demonstrate specific protection, there were antigen-specific immune responses and potential adjuvant effects by the innate response.

Baculovirus pseudotype viruses have also been explored. The G protein of vesicular stomatitis virus controlled by the insect polyhedron promoter and the HA of A/Chicken/Hubei/327/2004 (H5N1) HPAIV controlled by a CMV promoter were used to generate the BV-G-HA. Intramuscular immunization of mice or chickens with BV-G-HA elicited strong HI and VN serum antibody responses, IFN-γ responses, and protected from H5N1 challenge [79]. A separate study demonstrated efficacy using a bivalent pseudotyped baculovirus vector [80].

Baculovirus has also been used to generate an inactivated particle vaccine. The HA of A/Indonesia/CDC669/2006(H5N1) was incorporated into a commercial baculovirus vector controlled by the e1 promoter from White Spot Syndrome Virus. The resulting recombinant virus was propagated in insect (Sf9) cells and inactivated as a particle vaccine [81,82]. Intranasal delivery with cholera toxin B as an adjuvant elicited robust HI titers and protected from lethal challenge [81]. Oral delivery of this encapsulated vaccine induced robust serum HI titers and mucosal IgA titers in mice, and protected from H5N1 HPAIV challenge. More recently, co-formulations of inactivated baculovirus vectors have also been shown to be effective in mice [83].

While there is growing data on the potential use of baculovirus or pseudotyped baculovirus as a vaccine vector, efficacy data in mammalian animal models other than mice is lacking. There is also no data on the safety in humans, reducing enthusiasm for baculovirus as a vaccine vector for influenza at this time.

### 2.5. Newcastle Disease Virus Vectors

Newcastle disease virus (NDV) is a single-stranded, negative-sense RNA virus that causes disease in poultry. NDV has a number of appealing qualities as a vaccine vector. As an avian virus, there is little or no preexisting immunity to NDV in humans and NDV propagates to high titers in both chicken eggs and cell culture. As a paramyxovirus, there is no DNA phase in the virus lifecycle reducing concerns of integration events, and the levels of gene expression are driven by the proximity to the leader sequence at the 3' end of the viral genome. This gradient of gene expression enables attenuation through rearrangement of the genome, or by insertion of transgenes within the genome. Finally, pathogenicity of NDV is largely determined by features of the fusion protein enabling ready attenuation of the vaccine vector [84].

Reverse genetics, a method that allows NDV to be rescued from plasmids expressing the viral RNA polymerase and nucleocapsid proteins, was first reported in 1999 [85,86]. This process has enabled manipulation of the NDV genome as well as incorporation of transgenes and the development of NDV vectors. Influenza was the first infectious disease targeted with a recombinant NDV (rNDV) vector. The HA protein of A/WSN/1933 (H1N1) was inserted into the Hitchner B1 vaccine strain. The HA protein was expressed on infected cells and was incorporated into infectious virions. While the virus was attenuated compared to the parental vaccine strain, it induced a robust serum antibody response and protected against homologous influenza virus challenge in a murine model of infection [87]. Subsequently, rNDV was tested as a vaccine vector for HPAIV having varying efficacy against H5 and H7 influenza virus infections in poultry [88,89,90,91,92,93,94]. These vaccines have the added benefit of potentially providing protection against both the influenza virus and NDV infection.

NDV has also been explored as a vaccine vector for humans. Two NHP studies assessed the immunogenicity and efficacy of an rNDV expressing the HA or NA of A/Vietnam/1203/2004 (H5N1; VN1203) [95,96]. Intranasal and intratracheal delivery of the rNDV-HA or rNDV-NA vaccines induced both serum and mucosal antibody responses and protected from HPAIV challenge [95,96]. NDV has limited clinical data; however, phase I and phase I/II clinical trials have shown that the NDV vector is well-tolerated, even at high doses delivered intravenously [44,97]. While these results are promising, additional studies are needed to advance NDV as a human vaccine vector for influenza.

### 2.6. Parainfluenza Virus 5 Vectors

Parainfluenza virus type 5 (PIV5) is a paramyxovirus vaccine vector being explored for delivery of influenza and other infectious disease vaccine antigens. PIV5 has only recently been described as a vaccine vector [98]. Similar to other RNA viruses, PIV5 has a number of features that make it an attractive vaccine vector. For example, PIV5 has a stable RNA genome and no DNA phase in virus replication cycle reducing concerns of host genome integration or modification. PIV5 can be grown to very high titers in mammalian vaccine cell culture substrates and is not cytopathic allowing for extended culture and harvest of vaccine virus [98,99]. Like NDV, PIV5 has a 3'-to 5' gradient of gene expression and insertion of transgenes at different locations in the genome can variably attenuate the virus and alter transgene expression [100]. PIV5 has broad tropism, infecting many cell types, tissues, and species without causing clinical disease, although PIV5 has been associated with “kennel cough” in dogs [99].

A reverse genetics system for PIV5 was first used to insert the HA gene from A/Udorn/307/72 (H3N2) into the PIV5 genome between the hemagglutinin-neuraminidase (HN) gene and the large (L) polymerase gene. Similar to NDV, the HA was expressed at high levels in infected cells and replicated similarly to the wild type virus, and importantly, was not pathogenic in immunodeficient mice [98]. Additionally, a single intranasal immunization in a murine model of influenza infection was shown to induce neutralizing antibody responses and protect against a virus expressing homologous HA protein [98]. PIV5 has also been explored as a vaccine against HPAIV. Recombinant PIV5 vaccines expressing the HA or NP from VN1203 were tested for efficacy in a murine challenge model. Mice intranasally vaccinated with a single dose of PIV5-H5 vaccine had robust serum and mucosal antibody responses, and were protected from lethal challenge. Notably, although cellular immune responses appeared to contribute to protection, serum antibody was sufficient for protection from challenge [100,101]. Intramuscular immunization with PIV5-H5 was also shown to be effective at inducing neutralizing antibody responses and protecting against lethal influenza virus challenge [101]. PIV5 expressing the NP protein of HPAIV was also efficacious in the murine immunization and challenge model, where a single intranasal immunization induced robust CD8^+^ T cell responses and protected against homologous (H5N1) and heterosubtypic (H1N1) virus challenge [102].

Currently there is no clinical safety data for use of PIV5 in humans. However, live PIV5 has been a component of veterinary vaccines for “kennel cough” for >30 years, and veterinarians and dog owners are exposed to live PIV5 without reported disease [99]. This combined with preclinical data from a variety of animal models suggests that PIV5 as a vector is likely to be safe in humans. As preexisting immunity is a concern for all virus-vectored vaccines, it should be noted that there is no data on the levels of preexisting immunity to PIV5 in humans. However, a study evaluating the efficacy of a PIV5-H3 vaccine in canines previously vaccinated against PIV5 (kennel cough) showed induction of robust anti-H3 serum antibody responses as well as high serum antibody levels to the PIV5 vaccine, suggesting preexisting immunity to the PIV5 vector may not affect immunogenicity of vaccines even with repeated use [99].

### 2.7. Poxvirus Vectors

Poxvirus vaccines have a long history and the notable hallmark of being responsible for eradication of smallpox. The termination of the smallpox virus vaccination program has resulted in a large population of poxvirus-naïve individuals that provides the opportunity for the use of poxviruses as vectors without preexisting immunity concerns [103]. Poxvirus-vectored vaccines were first proposed for use in 1982 with two reports of recombinant vaccinia viruses encoding and expressing functional thymidine kinase gene from herpes virus [104,105]. Within a year, a vaccinia virus encoding the HA of an H2N2 virus was shown to express a functional HA protein (cleaved in the HA1 and HA2 subunits) and be immunogenic in rabbits and hamsters [106]. Subsequently, all ten of the primary influenza proteins have been expressed in vaccine virus [107].

Early work with intact vaccinia virus vectors raised safety concerns, as there was substantial reactogenicity that hindered recombinant vaccine development [108]. Two vaccinia vectors were developed to address these safety concerns. The modified vaccinia virus Ankara (MVA) strain was attenuated by passage 530 times in chick embryo fibroblasts cultures. The second, New York vaccinia virus (NYVAC) was a plaque-purified clone of the Copenhagen vaccine strain rationally attenuated by deletion of 18 open reading frames [109,110,111].

#### 2.7.1. Modified vaccinia virus Ankara (MVA) Vectors

Modified vaccinia virus Ankara (MVA) was developed prior to smallpox eradication to reduce or prevent adverse effects of other smallpox vaccines [109]. Serial tissue culture passage of MVA resulted in loss of 15% of the genome, and established a growth restriction for avian cells. The defects affected late stages in virus assembly in non-avian cells, a feature enabling use of the vector as single-round expression vector in non-permissive hosts. Interestingly, over two decades ago, recombinant MVA expressing the HA and NP of influenza virus was shown to be effective against lethal influenza virus challenge in a murine model [112]. Subsequently, MVA expressing various antigens from seasonal, pandemic (A/California/04/2009, pH1N1), equine (A/Equine/Kentucky/1/81 H3N8), and HPAI (VN1203) viruses have been shown to be efficacious in murine, ferret, NHP, and equine challenge models [113]. MVA vaccines are very effective stimulators of both cellular and humoral immunity. For example, abortive infection provides native expression of the influenza antigens enabling robust antibody responses to native surface viral antigens. Concurrently, the intracellular influenza peptides expressed by the pox vector enter the class I MHC antigen processing and presentation pathway enabling induction of CD8^+^ T cell antiviral responses. MVA also induces CD4^+^ T cell responses further contributing to the magnitude of the antigen-specific effector functions [107,112,113,114,115]. MVA is also a potent activator of early innate immune responses further enhancing adaptive immune responses [116]. Between early smallpox vaccine development and more recent vaccine vector development, MVA has undergone extensive safety testing and shown to be attenuated in severely immunocompromised animals and safe for use in children, adults, elderly, and immunocompromised persons. With extensive pre-clinical data, recombinant MVA vaccines expressing influenza antigens have been tested in clinical trials and been shown to be safe and immunogenic in humans [117,118,119]. These results combined with data from other (non-influenza) clinical and pre-clinical studies support MVA as a leading viral-vectored candidate vaccine.

#### 2.7.2. NYVAC Vectors

The NYVAC vector is a highly attenuated vaccinia virus strain. NYVAC is replication-restricted; however, it grows in chick embryo fibroblasts and Vero cells enabling vaccine-scale production. In non-permissive cells, critical late structural proteins are not produced stopping replication at the immature virion stage [120]. NYVAC is very attenuated and considered safe for use in humans of all ages; however, it predominantly induces a CD4^+^ T cell response which is different compared to MVA [114]. Both MVA and NYVAC provoke robust humoral responses, and can be delivered mucosally to induce mucosal antibody responses [121]. There has been only limited exploration of NYVAC as a vaccine vector for influenza virus; however, a vaccine expressing the HA from A/chicken/Indonesia/7/2003 (H5N1) was shown to induce potent neutralizing antibody responses and protect against challenge in swine [122]. 

While there is strong safety and efficacy data for use of NYVAC or MVA-vectored influenza vaccines, preexisting immunity remains a concern. Although the smallpox vaccination campaign has resulted in a population of poxvirus-naïve people, the initiation of an MVA or NYVAC vaccination program for HIV, influenza or other pathogens will rapidly reduce this susceptible population. While there is significant interest in development of pox-vectored influenza virus vaccines, current influenza vaccination strategies rely upon regular immunization with vaccines matched to circulating strains. This would likely limit the use and/or efficacy of poxvirus-vectored influenza virus vaccines for regular and seasonal use [13]. Intriguingly, NYVAC may have an advantage for use as an influenza vaccine vector, because immunization with this vector induces weaker vaccine-specific immune responses compared to other poxvirus vaccines, a feature that may address the concerns surrounding preexisting immunity [123].

#### 2.7.3. Veterinary Pox Vectors

While poxvirus-vectored vaccines have not yet been approved for use in humans, there is a growing list of licensed poxvirus for veterinary use that include fowlpox- and canarypox-vectored vaccines for avian and equine influenza viruses, respectively [124,125]. The fowlpox-vectored vaccine expressing the avian influenza virus HA antigen has the added benefit of providing protection against fowlpox infection. Currently, at least ten poxvirus-vectored vaccines have been licensed for veterinary use [126]. These poxvirus vectors have the potential for use as vaccine vectors in humans, similar to the first use of cowpox for vaccination against smallpox [127]. The availability of these non-human poxvirus vectors with extensive animal safety and efficacy data may address the issues with preexisting immunity to the human vaccine strains, although the cross-reactivity originally described with cowpox could also limit use.

### 2.8. Vesicular Stomatitis Virus Vectors

Influenza vaccines utilizing vesicular stomatitis virus (VSV), a rhabdovirus, as a vaccine vector have a number of advantages shared with other RNA virus vaccine vectors. Both live and replication-defective VSV vaccine vectors have been shown to be immunogenic [128,129], and like *Paramyxoviridae*, the *Rhabdoviridae* genome has a 3'-to-5' gradient of gene expression enabling attention by selective vaccine gene insertion or genome rearrangement [130]. VSV has a number of other advantages including broad tissue tropism, and the potential for intramuscular or intranasal immunization. The latter delivery method enables induction of mucosal immunity and elimination of needles required for vaccination. Also, there is little evidence of VSV seropositivity in humans eliminating concerns of preexisting immunity, although repeated use may be a concern. Also, VSV vaccine can be produced using existing mammalian vaccine manufacturing cell lines.

Influenza antigens were first expressed in a VSV vector in 1997. Both the HA and NA were shown to be expressed as functional proteins and incorporated into the recombinant VSV particles [131]. Subsequently, VSV-HA, expressing the HA protein from A/WSN/1933 (H1N1) was shown to be immunogenic and protect mice from lethal influenza virus challenge [129]. To reduce safety concerns, attenuated VSV vectors were developed. One candidate vaccine had a truncated VSV G protein, while a second candidate was deficient in G protein expression and relied on G protein expressed by a helper vaccine cell line to the provide the virus receptor. Both vectors were found to be attenuated in mice, but maintained immunogenicity [128]. More recently, single-cycle replicating VSV vaccines have been tested for efficacy against H5N1 HPAIV. VSV vectors expressing the HA from A/Hong Kong/156/97 (H5N1) were shown to be immunogenic and induce cross-reactive antibody responses and protect against challenge with heterologous H5N1 challenge in murine and NHP models [132,133,134].

VSV vectors are not without potential concerns. VSV can cause disease in a number of species, including humans [135]. The virus is also potentially neuroinvasive in some species [136], although NHP studies suggest this is not a concern in humans [137]. Also, while the incorporation of the influenza antigen in to the virion may provide some benefit in immunogenicity, changes in tropism or attenuation could arise from incorporation of different influenza glycoproteins. There is no evidence for this, however [134]. Currently, there is no human safety data for VSV-vectored vaccines. While experimental data is promising, additional work is needed before consideration for human influenza vaccination.

## 3. Universal Vaccines

Current influenza vaccines rely on matching the HA antigen of the vaccine with circulating strains to provide strain-specific neutralizing antibody responses [4,14,24]. There is significant interest in developing universal influenza vaccines that would not require annual reformulation to provide protective robust and durable immunity. These vaccines rely on generating focused immune responses to highly conserved portions of the virus that are refractory to mutation [30,31,32]. Traditional vaccines may not be suitable for these vaccination strategies; however, vectored vaccines that have the ability to be readily modified and to express transgenes are compatible for these applications.

The NP and M2 proteins have been explored as universal vaccine antigens for decades. Early work with recombinant viral vectors demonstrated that immunization with vaccines expressing influenza antigens induced potent CD8^+^ T cell responses [107,138,139,140,141]. These responses, even to the HA antigen, could be cross-protective [138]. A number of studies have shown that immunization with NP expressed by AAV, rAd5, alphavirus vectors, MVA, or other vector systems induces potent CD8^+^ T cell responses and protects against influenza virus challenge [52,63,69,102,139,142]. As the NP protein is highly conserved across influenza A viruses, NP-specific T cells can protect against heterologous and even heterosubtypic virus challenges [30].

The M2 protein is also highly conserved and expressed on the surface of infected cells, although to a lesser extent on the surface of virus particles [30]. Much of the vaccine work in this area has focused on virus-like or subunit particles expressing the M2 ectodomain; however, studies utilizing a DNA-prime, rAd-boost strategies to vaccinate against the entire M2 protein have shown the antigen to be immunogenic and protective [50]. In these studies, antibodies to the M2 protein protected against homologous and heterosubtypic challenge, including a H5N1 HPAIV challenge. More recently, NP and M2 have been combined to induce broadly cross-reactive CD8^+^ T cell and antibody responses, and rAd5 vaccines expressing these antigens have been shown to protect against pH1N1 and H5N1 challenges [29,51].

Historically, the HA has not been widely considered as a universal vaccine antigen. However, the recent identification of virus neutralizing monoclonal antibodies that cross-react with many subtypes of influenza virus [143] has presented the opportunity to design vaccine antigens to prime focused antibody responses to the highly conserved regions recognized by these monoclonal antibodies. The majority of these broadly cross-reactive antibodies recognize regions on the stalk of the HA protein [143]. The HA stalk is generally less immunogenic compared to the globular head of the HA protein so most approaches have utilized “headless” HA proteins as immunogens. HA stalk vaccines have been designed using DNA and virus-like particles [144] and MVA [142]; however, these approaches are amenable to expression in any of the viruses vectors described here.

## 4. Conclusions

The goal of any vaccine is to protect against infection and disease, while inducing population-based immunity to reduce or eliminate virus transmission within the population. It is clear that currently licensed influenza vaccines have not fully met these goals, nor those specific to inducing long-term, robust immunity. There are a number of vaccine-related issues that must be addressed before population-based influenza vaccination strategies are optimized. The concept of a “one size fits all” vaccine needs to be updated, given the recent ability to probe the virus–host interface through RNA interference approaches that facilitate the identification of host genes affecting virus replication, immunity, and disease. There is also a need for revision of the current influenza virus vaccine strategies for at-risk populations, particularly those at either end of the age spectrum. An example of an improved vaccine regime might include the use of a vectored influenza virus vaccine that expresses the HA, NA and M and/or NP proteins for the two currently circulating influenza A subtypes and both influenza B strains so that vaccine take and vaccine antigen levels are not an issue in inducing protective immunity. Recombinant live-attenuated or replication-deficient influenza viruses may offer an advantage for this and other approaches. 

Vectored vaccines can be constructed to express full-length influenza virus proteins, as well as generate conformationally restricted epitopes, features critical in generating appropriate humoral protection. Inclusion of internal influenza antigens in a vectored vaccine can also induce high levels of protective cellular immunity. To generate sustained immunity, it is an advantage to induce immunity at sites of inductive immunity to natural infection, in this case the respiratory tract. Several vectored vaccines target the respiratory tract. Typically, vectored vaccines generate antigen for weeks after immunization, in contrast to subunit vaccination. This increased presence and level of vaccine antigen contributes to and helps sustain a durable memory immune response, even augmenting the selection of higher affinity antibody secreting cells. The enhanced memory response is in part linked to the intrinsic augmentation of immunity induced by the vector. Thus, for weaker antigens typical of HA, vectored vaccines have the capacity to overcome real limitations in achieving robust and durable protection.

Meeting the mandates of seasonal influenza vaccine development is difficult, and to respond to a pandemic strain is even more challenging. Issues with influenza vaccine strain selection based on recently circulating viruses often reflect recommendations by the World Health Organization (WHO)—a process that is cumbersome. The strains of influenza A viruses to be used in vaccine manufacture are not wild-type viruses but rather reassortants that are hybrid viruses containing at least the HA and NA gene segments from the target strains and other gene segments from the master strain, PR8, which has properties of high growth in fertilized hen’s eggs. This additional process requires more time and quality control, and specifically for HPAI viruses, it is a process that may fail because of the nature of those viruses. In contrast, viral-vectored vaccines are relatively easy to manipulate and produce, and have well-established safety profiles. There are several viral-based vectors currently employed as antigen delivery systems, including poxviruses, adenoviruses baculovirus, paramyxovirus, rhabdovirus, and others; however, the majority of human clinical trials assessing viral-vectored influenza vaccines use poxvirus and adenovirus vectors. While each of these vector approaches has unique features and is in different stages of development, the combined successes of these approaches supports the virus-vectored vaccine approach as a whole. Issues such as preexisting immunity and cold chain requirements, and lingering safety concerns will have to be overcome; however, each approach is making progress in addressing these issues, and all of the approaches are still viable. Virus-vectored vaccines hold particular promise for vaccination with universal or focused antigens where traditional vaccination methods are not suited to efficacious delivery of these antigens. The most promising approaches currently in development are arguably those targeting conserved HA stalk region epitopes. Given the findings to date, virus-vectored vaccines hold great promise and may overcome the current limitations of influenza vaccines.
